# Significant Volume Expansion as a Precursor to Ablation and Micropattern Formation in Phase Change Material Induced by Intense Terahertz Pulses

**DOI:** 10.1038/s41598-018-21275-3

**Published:** 2018-02-13

**Authors:** Kotaro Makino, Kosaku Kato, Keisuke Takano, Yuta Saito, Junji Tominaga, Takashi Nakano, Goro Isoyama, Makoto Nakajima

**Affiliations:** 1 Nanoelectronics Research Institute, National Institute of Advanced Industrial Science and Technology (AIST), Tsukuba, Ibaraki 305-8565 Japan; 2 Institute of Laser Engineering (ILE), Osaka University, Suita, Osaka 565-0871 Japan; 3 Institute of Scientific and Industrial Research (ISIR), Osaka University, Ibaraki, Osaka 567-0047 Japan

## Abstract

With rapid advances occurring in terahertz (THz) radiation generation techniques, the interaction between matter and intense THz fields has become an important research topic. Among different types of THz radiation sources, the free electron laser (FEL) is a promising experimental tool that is expected to pave the way for new forms of material processing, control of phase transitions, and serve as a test bench for extreme operating conditions in high-speed small-size electrical and magnetic devices through the exploitation of strong THz electrical and magnetic fields without the presence of interband electronic excitation. In the current work, we irradiated Ge_2_Sb_2_Te_5_ phase change memory material with intense THz pulse trains from an FEL and observed THz-induced surface changes due to damage as a precursor to ablation and the formation of fine surface undulations whose spatial period is comparable to or slightly smaller than the wavelength of the excitation THz pulses in the material. The formation of undulations as well as the fact that no significant thermal effect was observed below the volume expansion threshold suggests that THz-induced effects mainly contributed to the observed changes. To the best of our knowledge, this is the first experimental observation of THz-induced undulations (so-called “*LIPSS*”), which are of potential importance for laser material processing.

## Introduction

Terahertz (THz) radiation, electromagnetic radiation whose oscillation frequency lies in the THz range, has photon energies in the milli-electron volt range and has been commonly used for spectroscopic measurements and imaging applications as insulators are essentially transparent to THz radiation whereas semiconductors and metals are opaque in the presence of free carriers^1^. Moreover, THz radiation can couple to molecular motion, phonons, and the spin degree of freedom. Recently, with the development of the current generation of intense THz radiation sources, intense and short THz pulses hold promise as a tool for fast material manipulation via THz-matter interaction without the presence of interband electronic excitations. Control of material structure by the direct manipulation of atomic and lattice motions and electronic state by application of intense electrical fields that can lead to the instantaneous acceleration of electrons, impact ionization, and insulator breakdown, as well as modification of the spin degrees of freedom by THz magnetic fields can be realized with intense THz pulses. The control of orbital order in strongly correlated material^2^, changes in polymer morphology^3^, and unique field electron emission processes^4^ have been reported using THz free electron laser (FEL) pulses. However, intense THz FEL pulses have not yet been applied to laser material processing techniques such as cutting, marking, ablation, and micropatterning. As observed in the previous studies described above, intense THz pulses can lead to unique effects in matter and hence the exploitation of an FEL is expected to pave the way for a new class of laser processing applications.

At the same time, intense THz radiation can be used as a test tool for high-speed electrical and magnetic device studies. Use of THz pulses goes beyond the limits of conventional measurements, enabling the application of instantaneous electrical and magnetic fields to a material or a device and can be used to replicate extreme operating conditions in high-speed electrical and magnetic devices in which their operation is induced by instantaneous high-intensity electrical or magnetic fields. For electrical phase change memory^5^, the memory operation is governed by the application of a voltage pulse to a phase change cell; thus, the instantaneous application of an electrical field is an important research area for high-speed operation and the understanding of the phase change process. Indeed, Zalden *et al*. have demonstrated THz-induced sub-picosecond threshold switching from the amorphous phase to the crystalline phase in amorphous Ag-In-Sb-Te phase change memory material at rates beyond the speed limit of nucleation, which indicates the possibility of ultrafast phase change operation^6^.

Here, we report upon the effect of intense THz pulses derived from an FEL on two different crystalline Ge_2_Sb_2_Te_5_ (GST) phase change material samples with different electrical resistivities. Among the various types of THz sources, an FEL can be used to generate a very intense THz pulse train with narrow band and fine frequency tunability. GST has been widely utilized for applications in nonvolatile electrical memory as well as in rewritable optical discs^7^. The SET-RESET memory operation, also known as crystalline-amorphous phase transition, is realized by application of electrical or optical pulses that induce thermal annealing or melt-quench disordering effects on the structure of GST. Since this structural phase transition results in significant differences in electrical resistivity and optical reflectivity, data storage can be realized. With increasing temperature, amorphous (RESET) GST transforms into the cubic and hexagonal crystalline (SET) phases at $\simeq$150 and $\simeq$350 °C, respectively. At the same time, an insulator-to-metal transition occurs and both a significant electrical resistance decrease^8^ and an optical reflectivity increase^9^ are induced depending on temperature. On the other hand, the SET phases transform into the RESET phase via a molten state by a melt and quench procedure.

The samples investigated consisted of cubic GST films with different electrical resistances deposited on high-resistance Si substrates by sputtering. The electrical resistance values between two contacts separated by 1 cm were ~110 kΩ and ~18 kΩ, leading to the labeling of the samples as *high*-*R* and *low*-*R* GSTs, respectively; the resistance (conductivity) of the *high*-*R* GST sample was six times (one-sixth) as large as that of the *low*-*R* GST sample predominately due to a change in the carrier mobility^10^.

**Figure 1:**
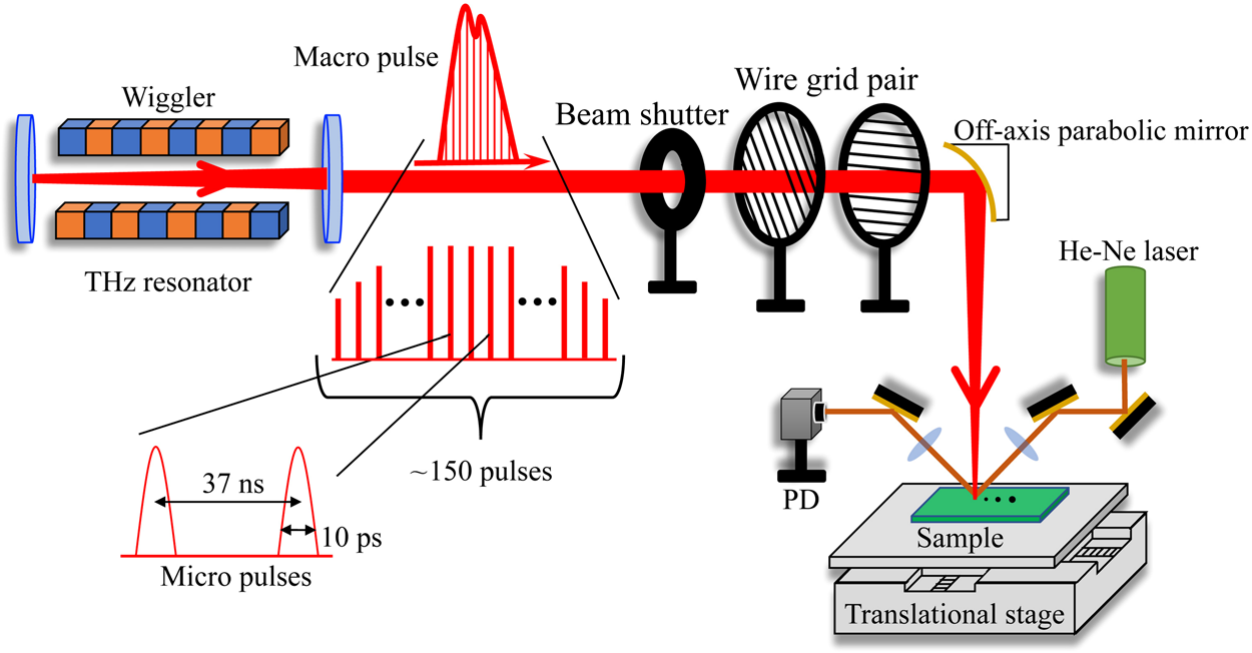
Experimental setup. An intense narrow-band THz pulse is generated with a high-energy electron pulse accelerated by a linear accelerator in a free-electron laser, which consists of a horizontally oscillation type wiggler and an optical cavity with a coupling hole from the upstream mirror. The FEL was operated at 0.2 Hz to produce a train of ~150 micropulses of ~10 ps duration at 37 ns intervals, which is referred to as a macropulse. A pair of wire grid polarizers was employed to control of the THz pulse power. THz pulses were focused onto the sample surface at normal incidence. To monitor the reflectivity change of THz-irradiated area, a 633 nm He-Ne laser was used. A loosely focused He-Ne laser beam was overlapped with the THz spot on the sample surface. The transient intensity of the reflected He-Ne beam was measured with a photo detector (PD). The sample was moved stepwise after each macropulse shot.

Figure 1 shows a schematic illustration of the experimental setup. The FEL emits intense THz macropulses which consist of a train of $\simeq$150 micropulses with a 4 THz centre frequency, a 10 ps duration, and a 37 ns interval between individual micro pulses. Macropulses from the FEL were focused by an off-axis parabolic mirror onto the sample surface under normal incident conditions. A pair of wire grid polarizers was used to control the intensity of the THz pulse and a beam shutter was used to carry out single pulse train measurements. We employed a continuous wave He-Ne laser to monitor the reflectivity change in the THz-irradiated area of the sample. The He-Ne light was directed and loosely focused onto the sample surface by a lens at the same position of the incident THz pulse and the reflected light was detected by a photo diode (PD). The spot size of the He-Ne light was arranged to be larger than that of the THz pulse to avoid thermal effects due to the He-Ne light. The samples were placed on a translational stage and were translated to expose a new sample area after each shot of macropulse. We confirmed that a bare Si substrate is not affected by THz pulse excitations for any fluence used in this experiment.

## Results

**Figure 2:**
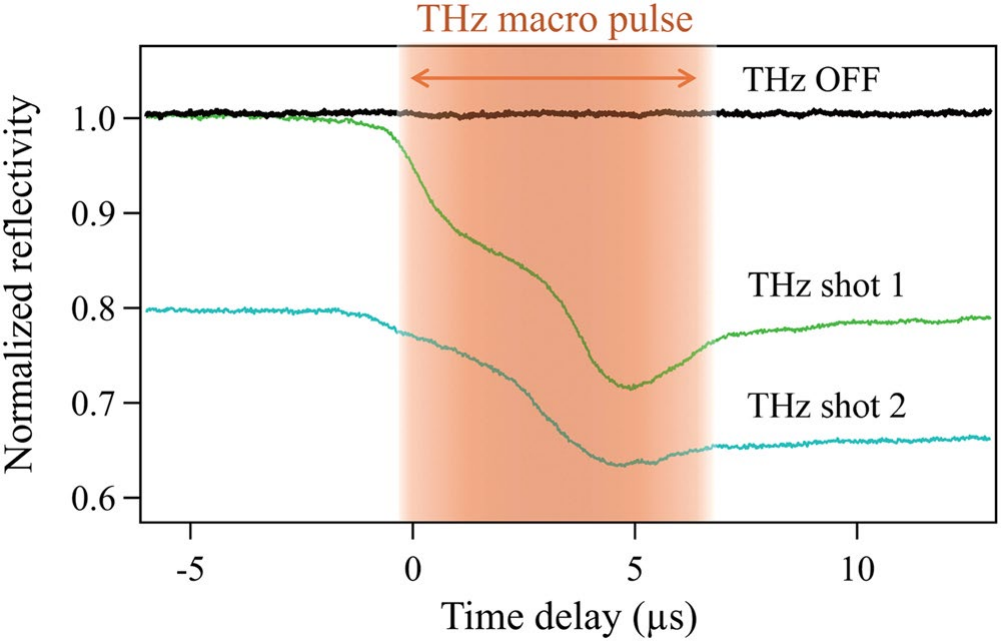
Time-resolved reflectivity change of the THz-irradiated area. The intensity of the He-Ne laser signal reflected at the THz-irradiated spot as a function of time for the *high*-*R* GST sample for *F* = 1.0 J/cm^2^. A similar signal was also obtained for the *low*-*R* GST sample. The intensity was normalized based on the He-Ne intensity before THz excitation. The zero for the time delay was determined by trigger signals provided by the FEL facility. The samples were irradiated with two temporally separated THz pulses each separated by more than 10 seconds, with the reflectivity measured at the same position. The temporal duration of the THz macro pulse is schematically shown by the light brown area.

Figure 2 shows the typical time-dependent He-Ne laser reflectivity change observed for the *high*-*R* GST sample surface induced by two consecutive THz macropulse shots with an intensity above the colour change threshold (described later) at the same position. A very similar behaviour was observed for the *low*-*R* GST sample. The constant signal intensity observed before the THz excitation demonstrates that the He-Ne laser does not induce any thermal effects. The reflectivity started to drop continuously after the arrival of the first THz pulse. One can see two inflection points during the passage of macropulse through the sample. After passage of the first pulse, the reflectivity slightly recovered and reached to a constant value before the arrival of the second pulse after a delay of more than ten seconds. This static reduction in the reflectivity after the first THz pulse excitation suggests that a permanent change in the reflectivity of the sample surface was induced by the THz pulse. During the THz macropulse excitation, the reflectivity transiently dropped below the level obtained after the first pulse passed; hence, the samples are expected to experience a transient THz-excited state in which the reflectivity is modified due to the presence of a THz-induced plasma and a volume expansion caused by an increase in temperature. The second shot of the THz macropulse induced a similar effect, leading to a small difference in reflectivity. The shape of the curves is thought to partially correspond to the temporal intensity profile of the THz macropulses^4^. It should be noted that the actual reflectivity change in the THz-excited area is larger than the experimental value because the probe beam spot is large compared with the THz-irradiated area.

**Figure 3:**
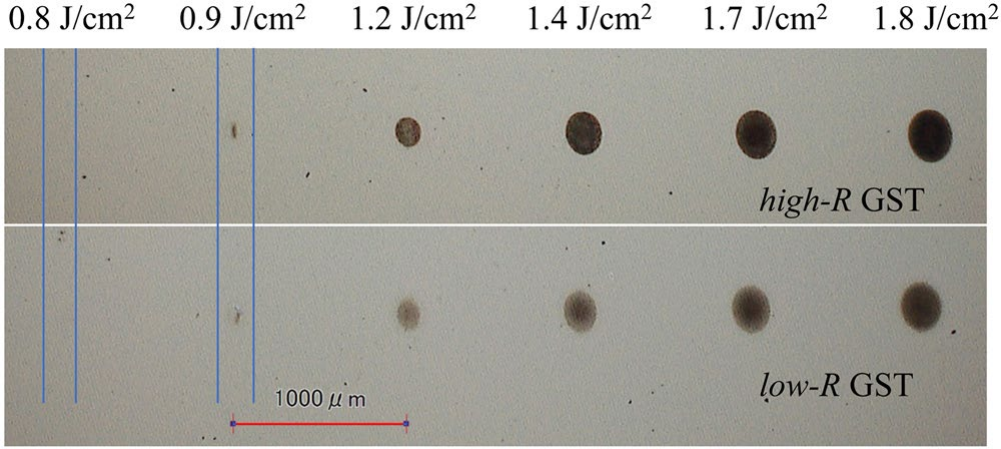
Variation of THz-induced marks for the *high*-*R* and *low*-*R* GST samples. THz-induced marks obtained by a single macropulse for various fluences for the *high*-*R* and *low*-*R* GST samples. The irradiation position was varied by 1.0 mm after each THz macropulse. Below *F* = 0.8 J/cm^2^, no significant change was observed. At *F* = 1.8 J/cm^2^, a black pattern emerged, with the pattern gradually enlarged for increasing THz fluence.

To document the THz-induced change of the samples, we observed the surface colour changes for the samples using a digital microscope. For this measurement, we irradiated both the *high*-*R* and *low*-*R* GST samples with a single THz macropulse. The fluence of the THz pulse (*F*) was varied from 0.5 to 1.8 J/cm^2^ (from 0.3 to 1.2 GW/cm^2^ in intensity). Here, *F* indicates the normalized fluence value within the Gaussian-shapes focused THz spot diameter at the sample position. In the THz spot, the local fluence reaches a peak at the centre and decreases with increasing distance from the centre. The sample stage was moved 1 mm after each shot to evaluate the effect of a single THz macropulse. In order to avoid the extrinsic effects caused by the He-Ne light excitation, we turned off the He-Ne laser beforehand. Figure 3 shows microscope images of the THz-irradiated areas for the *high*-*R* and *low*-*R* GST samples. We observed similar behaviour for the THz-induced changes in both samples as follows: Below *F* = 0.8 J/cm^2^, no significant colour change was observed. At *F* = 0.9 J/cm^2^, we found discoloured marks in the THz-irradiated area; the formation of the THz-induced mark is thought to be a threshold phenomenon. With increasing THz pulse fluence up to *F* = 1.8 J/cm^2^, the diameter of the mark increased, and its colour became darker. Note that the difference in the slight background (outside of the THz-irradiated area) brightness for the samples indicates a difference in the reflectivity due to the different electrical properties of the *high*-*R* and *low*-*R* GST samples as *low*-*R* GST shows more metallic features and therefore exhibits a higher reflectivity than *high*-*R* GST^9^.

**Figure 4:**
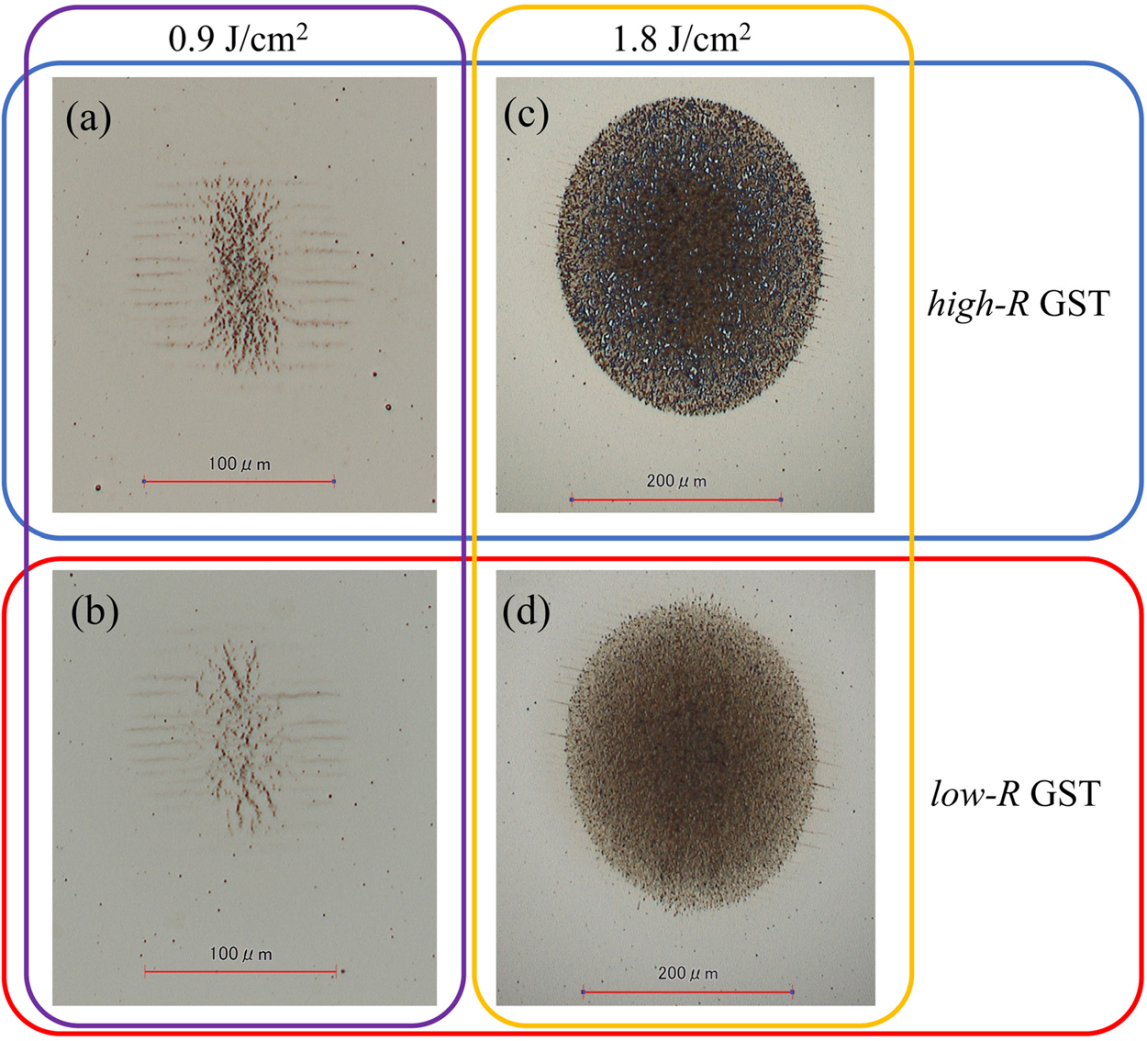
Enlarged microscope images of the THz-irradiated area. Magnified view of the THz-induced marks occurring for at *F* = 0.9 J/cm^2^ and 1.8 J/cm^2^ for *high*-*R* and *low*-*R* GST samples. For all the marks, a black region and surrounding black undulations can be seen.

Figure 4 shows magnified images of the THz-irradiated area for *high*-*R* and *low*-*R* GST samples caused by a single macropulse shot with a fluence of 0.9 J/cm^2^ and 1.8 J/cm^2^ fluences. It is remarkable that the THz-induced colour changes of the films are not uniform but mainly consist of two different regions as shown in Fig. 4(a,c). The irradiated area is characterized by an elliptically distributed black area surrounded by an undulation pattern in which approximately parallel black lines are aligned along the horizontal direction of the sample. Here, the horizontal direction corresponds to the polarization direction of THz pulse. We confirmed that the direction of undulations was always roughly parallel to the THz pulse polarization and does not depend on the orientation of the sample. The presence of a ZnS-SiO_2_ protection layer itself can be excluded from being the main origin for the observed undulations because no undulation pattern was observed for another GST sample (Supplementary Fig. S1) although the interface between the protection layer and the GST might have contributed to the result. Since the spatial distribution of the THz fluence is Gaussian, we found that the formation of the center black area requires higher fluence than that of the more distant undulations. In other words, a THz-induced colour change is formed at two threshold fluences that correspond to the formation of an undulation pattern (*F*_*undulation*_) and the centre black region (*F*_*black*_), and the *F*_*black*_ is higher than *F*_*undulation*_.

As can be seen in Fig. 4(c,d), with increasing THz fluence, the centre black region is enlarged but the surrounding undulated region shifts outward, with its width remaining almost constant. This feature is consistent with the model described above as increases in THz fluence simply enhance the local THz fluence without changing the spatial profile at the sample position and the size of the high fluence area whose local fluence is higher than *F*_*black*_ expands but the intermediate fluence area where the local fluence is between *F*_*black*_ and *F*_*undulation*_ just shifts outward. The undulations become slightly tilted when relatively intense THz pulse are incident as shown in Fig. 4(c,d). This tilt of the undulations is thought to be related to the spatial distribution of the polarization state. From Fig. 4(a), *F*_*undulation*_ and *F*_*black*_ are estimated to be 1.60 and 1.83 J/cm^2^, respectively, assuming a Gaussian spatial profile for the incident THz pulses.

**Figure 5:**
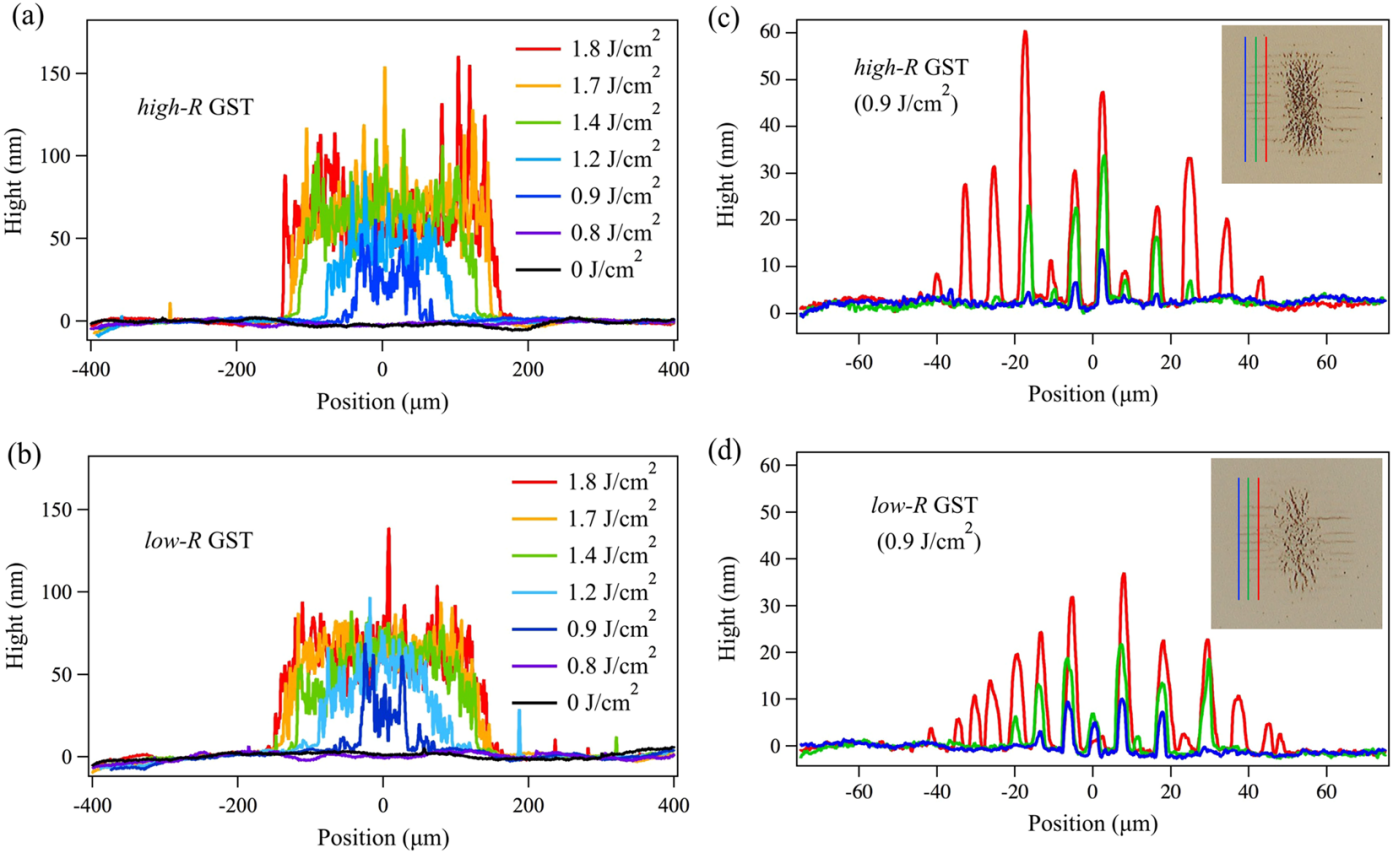
Surface profiles of THz-irradiated areas. (**a**,**b**) Fluence-dependent surface profiles of THz-induced marks measured along the vertical direction from the bottom to the top of Fig. 4 via the central THz irradiated region for the *high*-*R* and *low*-*R* GST samples. (**c**,**d**) Position-dependent profiles of the undulations (*F* = 0.9 J/cm^2^) measured along the lines shown in the inset. The colours of the lines in the plots correspond to the measurement traces in the insets. All profiles were measured by a stylus profiler.

The surface profiles for the THz-irradiated areas were also measured with a stylus depth profiler. Figure 5(a,b) show the THz fluence dependence of the surface profiles measured from the outside of the THz-marked area to the opposite side via the central black area. For both samples, a similar fluence dependence was observed. Below *F* = 0.8 J/cm^2^, we confirmed no significant change was introduced to the surface. At *F* = 0.9 J/cm^2^, an expansion of the surface was clearly observed. With increasing THz fluence, the height and size of the THz-induced area increased. The size of the expanded area for each fluence remained almost the same as that of the corresponding colour change. The observed change in the height of 20~100 nm is relatively large compared with the thickness of the GST film and the protection layer (60 nm in total). It should be emphasized that for GST, the THz-induced change is completely different from the “RESET phase change” of GST. When RESET phase change is induced, the expected volume expansion is much smaller than the current experimental result^11^. Therefore, we attribute the THz-induced change to a volume expansion due to a form of destruction of the structure that has not yet been observed by any other methods. Inside of the black area, the surface is rough and there are many spike-like features regardless of the position although the height of the expansion depends on the fluence. The surface profile trend reflects the fact that when the local fluence is higher than *F*_*black*_, an approximately similar roughness can be induced. On the other hand, the base height of the black area gradually increases with increasing THz fluence and saturates at *F* = 1.4 J/cm^2^ in both samples.

The surface profiles of the surrounding undulated areas were also measured. Figure 5(c,d) show position-dependent surface profiles of the THz-induced undulated areas (*F* = 0.9 J/cm^2^) for *high*-*R* and *low*-*R* GST samples, respectively. The profiles were measured along the three different traces which are approximately indicated by the corresponding colour lines in the inset of Fig. 5(c,d). These measurements revealed that the lines are also formed by the volume expansion and that their height depends on the local fluence in both the horizontal and vertical directions. The height and the number of peaks increased in the horizontal direction with increasing distance from the edge (blue lines) towards the interior (green and red lines). In the vertical direction, the height of the peaks tended to reach a maximum near the centre for all measurement traces. Therefore, the distribution of the line height is determined by the local fluence, independently of the direction of the lines. The spatial period (4~11 *μ*m) and the width of the undulations (2~4 *μ*m) are much smaller than the wavelength of the THz pulse (75 *μ*m), indicating the sub-wavelength features for the pattern, as will be discussed later.

## Discussion

As previously mentioned, the origin of the THz-induced black marks was found to be mainly the result of a significant volume expansion, which is a different phenomenon from the RESET phase change in the context of phase change materials. Instead of RESET phase change, a plausible reason for the volume expansion is likely to be a precursor of ablation, namely a kind of laser-induced damage^12^ or a destruction of the crystal structure (sort of amorphization that is different from RESET phase change). Due to the presence of a ZnS-SiO_2_ protection layer, the ablated GST might not be removed, which can lead to significant volume expansion. In general, the process of laser-pulse-driven ablation and damage is complicated and is an important topic of study in ultrafast laser science. A key condition for the occurrence of laser ablation and damage is an increase in the lattice temperature, that leads to the ejection of atoms, molecules, and clusters in the presence of bond softening and the formation of plasma arising from electronic excitation. When an ultrafast laser pulse (typically with sub-picosecond duration) is used, the electronic temperature instantaneously increases followed by a subsequent lattice temperature increase because the optical excitation initially excites the electronic system and its energy transfers thereafter to the lattice system on a picosecond time scale. In this situation, an imbalance between electronic and lattice energy drives non-thermal effects leading to ablation and damage. On the other hand, when the time period of a photoexcitation is longer than the time scale for energy transfer from photoexcited electrons to the lattice system, thermal effects dominantly work. In the current experiment, the pulse width of the micropulse is ~10 ps and that of the macropulse is ~6 *μ*s, and therefore mainly thermal effects should be taken into consideration.

Nevertheless, we found definite evidences for the THz-induced effects in addition to the thermal effects, as discussed below, even though it is difficult to directly estimate the THz-induced temperature increase due to the complicated temporal shape of the macropulse. If the volume expansion is mainly caused by thermal effects, the reflectivity change should occur for incident intensities below the ablation and damage thresholds: e.g., crystallization (RESET phase change) leads to brighter (darker) region on the *high*-*R* GST samples. However no such effects were observed when the fluence was below the threshold for the THz-induced volume expansion as shown in Fig. 3. Furthermore, neither colour nor brightness changes also could be observed outside of the THz-induced marks. The similar fluence dependence of the THz-induced marks observed for both the *high*-*R* and *low*-*R* GST samples provides a clue to a better understanding of the phenomenon. Under the same excitation conditions, an increase in lattice temperature can be considered be proportional to the conductance of the samples^13,14^. Thus the estimated temperature rise induced in the *low*-*R* GST sample is expected to be significantly larger than that in the *high*-*R* GST sample. Note that this estimate is consistent with the previous THz studies in which significant annealing-induced THz absorption increases were observed in GeTe and GST^15,16^. It should be mentioned that the reflectivity is also expected to be slightly enhanced in concurrence with the increase in the absorption. This effect is negligible since both absorbed and transmitted THz fields are thought to affect on the samples and the reflectivity is much lower than the transmittance^15,16^. Nonetheless, the trend observed for the THz-induced mark formation was similar for both samples. We ascribed the absence of a thermal phase change and the conductance dependence to impact ionization. Under intense electrical fields, carrier multiplication due to impact ionization occurs, with the number of carriers increasing nonlinearly sometimes beyond the number of inherent carriers in narrow band-gap semiconductors^17–20^, leading to an additional increase in electrical conductance. This THz-induced nonlinear change in conductivity and the resulting nonlinear lattice temperature increase are expected to lead to the volume expansion in the absence of thermal phase change and lead to an indistinct conductance dependence.

The formation of an undulation pattern that cannot be explained by thermal effects^21^ provides distinct evidence of THz-induced effects. Indeed, the undulation patterns, also known as laser-induced periodic surface structure (LIPSS), have been observed in various types of materials exposed to multi-shot of visible and near-infrared optical pulses whose pulse fluence is slightly below the ablation threshold and is recognized as a signature of laser-induced coherence effects. LIPSS is typically categorized into two types with low-spatial-frequency LIPSS (LSFL) and high-spatial-frequency LIPSS (HSFL) depending on the size of the structure in comparison to the excitation laser wavelength^21–23^. The spatial periods for LSFL are close to the wavelength of the excitation pulse. On the other hand, HSFL is dominantly observed in transparent materials with relatively short pulses (typically with femtosecond duration), and its spatial period is sufficiently smaller than the excitation laser wavelength^24,25^. Given the relatively high refractive index of cubic GST (~8)^26^ in the THz frequency range, the effective wavelength at the sample is estimated to be ~9 *μ*m; thus, the THz-induced undulations can be classified as LSFL. In general, the orientation of the LSFL depends on the size of the band gap^23,27–31^. In wide band gap materials, the LSFL orientation can be parallel to the polarization of the first pulse of the excitation pulse sequence^23,31^. Despite the fact that GST is a narrow gap semiconductor, the band gap energy is much larger than the photon energy of the THz pulse train and the current result with respect to the direction of the undulation pattern is consistent with the previous reports. Note that a completely different type of THz-induced mark without undulations was observed at the surface of a multi-layered GST film in which thermal effect are expected to be more significant than those in the cubic GST samples due to its lower electrical resistance (Supplementary Fig. S1). This result implies that thermal effects do not lead to the formation of undulations by causing destruction of the film under the current experimental conditions. The competition between thermal and laser-induced coherent effects i s thought to govern the formation of the observed undulations.

Among the proposed mechanisms for the formation of LIPSS, the excitation of surface plasmon-polaritons^32–34^ is thought to play a dominant role in the formation of THz-induced undulations in addition to impact ionization since surface plasmon-polariton can be excited by intraband THz excitations in both the *high*-*R* and *low*-*R* GST samples as they are both p-type semiconductors^35^. Compared with visible and near-infrared optical pulses, the spot size of the focused THz pulse is large and thereby use of the intense THz pulses enables one to realize intriguing phenomena over large areas. The utilization of an intense THz pulse train can be a promising experimental tool to understand the physical background of LIPSS since by taking advantage of the long wavelength, a large LIPSS structure can be formed compared to that obtained by using visible and near-infrared pulses. The color of the THz-induced marks for the *low*-*R* GST sample were darker than those of *high*-*R* GST sample (Figs 3 and 4). Although the origin of this difference cannot be resolved by a profile measurement, the size of the grains inside the THz-induced marks is thought to be related to the colour as shown in Fig. 3(c,d). Perhaps formation of the small grain structures is sensitive to the position of the sample^36^ or the thermal effect that is more significant in *low*-*R* GST than in *high*-*R* GST. In general, the formation of LIPSS requires multiple-shot of pulses yet laser-induced ablation and damage do not. In this sense, it is considered that the LIPSS was formed posterior to or in synchronization with the volume expansion within the pulse train excitation. For a comprehensive understanding, measurements based on single and few micropulse shots^37^ are important topics for future studies.

From the point of view of electrical phase change memory in which the phase change operation is governed by an electrical pulse, the current results indicate that just simple application of a strong 10 ps electrical pulse train cannot be used to induce RESET phase change in cubic GST under the current experimental conditions. This is consistent with a previous study in which it was revealed that an incubation field is necessary to increase the phase change speed and realize sub-nanosecond operation^38^. Even though the threshold for the RESET phase change is nearly half of the ablation threshold fluence for optical pulses^39^, no trace of RESET phase change could be seen at any fluence. The lack of a RESET phase change can be plausibly explained by the fact that THz excitation cannot induce photoexcited states, which may play an important role in the amorphization process^40^. Compared with the previously reported ablation thresholds for femtosecond and nanosecond laser pulses^39,41^, the threshold value for the volume expansion induced by a microsecond THz macro pulse was found to be large. This difference can be understood by taking into account the high transmittance for THz radiation and heat transfer into the substrate at time scales faster than the period of the THz macro pulse. Single micropulse measurements are also considered to be desirable to clarify these points.

## Conclusion

We irradiated crystalline cubic Ge_2_Sb_2_Te_5_ phase change material samples with strong THz pulse trains generated by means of a free electron laser. A significant THz-induced decrease in the reflectivity was observed when the fluence of the THz macropulse was higher than the threshold value. It was found that a significant volume expansion due to a precursor to ablation occurred at the centre of the THz-irradiated area without undergoing a RESET phase change. Furthermore, a fine undulation pattern was also observed outside of the area experiencing a volume expansion. Surface profile measurement revealed that the size and height of the expanded area were enhanced with increasing THz fluence. Additionally, the height of the undulation pattern was found to depend on the local fluence. Although we used two samples with different electrical resistivities, very similar results were obtained for both samples. We conclude that THz-laser-induced effects due to carrier multiplication introduced by impact ionization and surface plasmon polaritons contributed to the observed conductance-independent volume expansion and the formation of undulations. The absence of a RESET phase change reflects the fact that electrically and photoexcited states play an important role in the RESET phase change process. Since THz pulse leads to unique excitation behaviour that is different from the optical-excitation process, the use of intense THz pulses is promising for the further development of laser processing techniques, including laser ablation and the formation of micro patterns.

## Methods

### GST samples

40-nm-thick Ge_2_Sb_2_Te_5_ samples were grown by RF-magnetron sputtering at room temperature on the 525-*μ*m-thick high-resistance Si (100) substrates with an electrical resistance of more than 10000 Ω · cm. 20-nm-thick ZnS-SiO_2_ protection layers were deposited on top of the sample without breaking the vacuum after the GST deposition to prevent oxidation. The as-grown samples were confirmed to be amorphous by X-ray diffraction (XRD) measurements and electrical resistance measurements. Subsequently, the samples were annealed at 210 and 300 °C for 30 minutes to obtain the *high*-*R* and *low*-*R* cubic GST samples.

### Free electron laser

The free electron laser facility at the Research Laboratory for Quantum Beam Science, attached to the Institute of Scientific and Industrial Research (ISIR), Osaka University was used^42,43^. The FEL consists of a 40 MeV L-band linear accelerator, a permanent magnet wiggler with a period length of 6 cm and 32 periods, and a 5.53 m long optical resonator. A multi-bunch electron beam is accelerated by the accelerator, which is then passed through the wiggler, in which a THz pulse in the optical resonator is amplified repeatedly for lasing. The intense THz pulse train generated is referred to as a macropulse and is composed of ~150 micropulses of ~10 ps duration at 37 ns interval, which is obtained from the optical resonator through a hole at the centre of the upstream mirror of the resonator. The FEL was operated at a center frequency of 4 THz with a ~1 THz bandwidth and a repetition rate of 0.2 Hz. The energy of the macropulse was measured with a thermal energy meter (Coherent J-25MB-LE). The standard deviation for the laser energy was less than 5%. The spatial width (1/*e*^2^) of the focused THz pulse was measured using a pinhole^44^ and was found to be ~0.69 mm. The samples were irradiated with a single shot of macropulse shot by means of a beam shutter.

## Electronic supplementary material

Supplementary Information
