# Supplementary material for: Significant Volume Expansion as a Precursor to Ablation and Micropattern Formation in Phase Change Material Induced by Intense Terahertz Pulses
Source: Sci Rep. 2018 Feb 13;8:2914. doi: 10.1038/s41598-018-21275-3 (PMC5811531; doi:10.1038/s41598-018-21275-3)
Supplement: Supplementary file 1 — Supplementary Information [file 41598_2018_21275_MOESM1_ESM.pdf]

Supplementary Information

Volume expansion and Micro Pattern Formation on Phase Change Material Induced by Intense Terahertz Pulse

Kotaro Makino[1], Kosaku Kato[2], Keisuke Takano[2], Yuta Saito[1], Junji Tominaga[1], Takashi Nakano[1], Goro Isoyama[3], and Makoto Nakajima[2]

[1]Nanoelectronics Research Institute, National Institute of Advanced Industrial Science and Technology (AIST), Tsukuba, Ibaraki, 305-8562, Japan

[2]Institute of Laser Engineering (ILE), Osaka University, Suita, Osaka, 565-0871, Japan

[3]Institute of Scientific and Industrial Research (ISIR), Osaka University, Ibaraki, Osaka, 567-0047, Japan

*k-makino@aist.go.jp

Supplementary Figure S1.

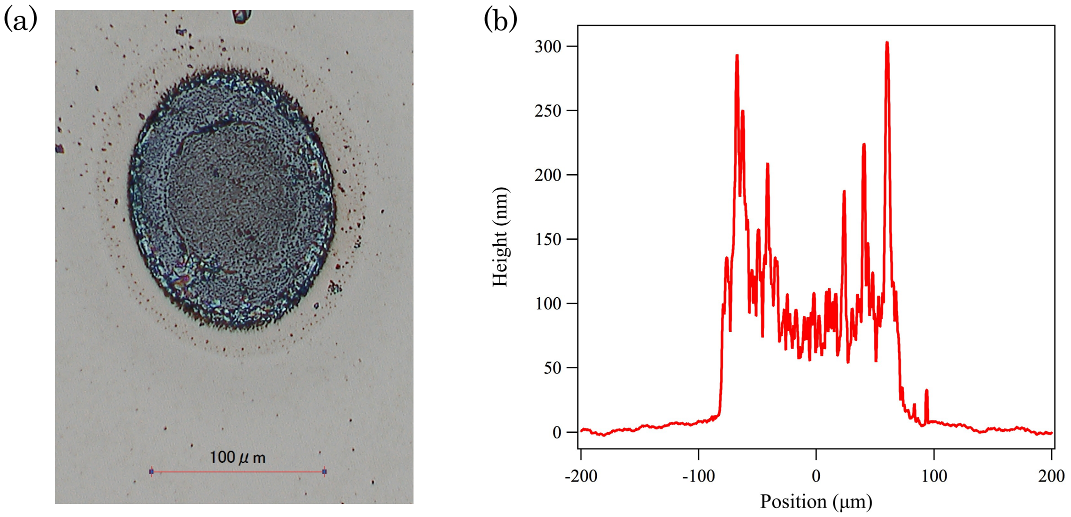

Figure S1. Digital microscope image (a) and surface profile (b) of THz-irradiated area (F = 1.2 J/cm^2) in multi-layered GST[1] ([$(GeTe)_2(Sb_2Te_3)_1]_{20}$ (40 nm) on the Si substrate measured in the same manner as Fig. 4 and Fig. 5, respectively. The sample is covered with a 20-nm-thick ZnS-SiO_2 protection layer. The electrical resistance between two contacts separated by 1 cm was 3.5 kΩ. Different from the results obtained in the cubic GST samples, a decrease in reflectivity was observed around the circular black mark. It was found that the expanded area just corresponds to the black mark. Judging by the

reflectivity loss and the lack of volume expansion, we attributed this surrounding low-reflectivity area to amorphous phase.

References

1. Simpson, R. E. *et al.* Interfacial phase-change memory. Nature Nanotech. **6**, 501–505 (2011).
